# DNA methylation-based ageing in a deuterostome invertebrate: an epigenetic clock for the crown-of-thorns seastar (*Acanthaster* cf. *solaris*)

**DOI:** 10.1098/rsbl.2026.0265

**Published:** 2026-09-09

**Authors:** Sarah L. T. Kwong, Alyssa Maree Budd, Maria Gomez-Cabrera, Julia Y.-H. Hung, Morgan S. Pratchett, Cecilia Villacorta-Rath, Sven Uthicke

**Affiliations:** ^1^ Australian Institute of Marine Science, PMB 3 Townsville MC, Townsville, Queensland, Australia; ^2^ College of Science and Engineering, James Cook University, Townsville, Queensland, Australia; ^3^ Centre for Tropical Bioinformatics and Molecular Biology, James Cook University, Townsville, Queensland, Australia; ^4^ Centre for Tropical Water and Aquatic Ecosystem Research (TropWATER), James Cook University, Townsville, Queensland, Australia; ^5^ Environomics Future Science Platform, Indian Ocean Marine Research Centre, Commonwealth Scientific and Industrial Research Organisation, Perth, Western Australia, Australia; ^6^ School of Molecular and Life Sciences, Curtin University, Perth, Western Australia, Australia; ^7^ UWA Oceans Institute, The University of Western Australia, Perth, Western Australia, Australia

**Keywords:** epigenetics, DNA methylation, molecular ageing tool, ageing biomarker, coral reef management

## Abstract

Accurate and reliable ageing tools are essential for wildlife conservation and management. While DNA methylation has emerged as a promising tool for age estimation in vertebrates, its application to invertebrates remains contested and has been limited to arthropods. Here, we develop an epigenetic clock for the Pacific crown-of-thorns seastar (CoTS; *Acanthaster* cf. *solaris*), a destructive coral predator contributing to habitat degradation across Indo-Pacific reefs. Using Oxford Nanopore Technologies, we generated whole-genome DNA methylation profiles across five age groups and identified 1910 CpG sites with methylation patterns significantly associated with age. We then fitted age prediction models using elastic net regression and evaluated predictive performance with leave-one-out cross-validation (LOOCV), achieving a mean absolute error of 0.31 ± 0.22 years, corresponding to 4–6% of the CoTS lifespan (5–8 years). This accuracy suggests the potential to differentiate annual cohorts, supporting future management-relevant inference. To facilitate practical implementation, we constructed an optimized epigenetic clock from 14 CpG sites consistently selected across LOOCV iterations. Our results demonstrate that DNA methylation-based age estimation is feasible in a deuterostome invertebrate, extending epigenetic ageing approaches beyond arthropods and establishing their potential to advance age determination and management in invertebrates that lack reliable ageing methods.

## 1. Introduction

Accurate age information is fundamental to ecological studies and species management, as it informs key life-history parameters such as growth and reproduction that underpin population models [1]. Yet, age determination in wildlife remains a significant challenge. In recent years, epigenetic ageing based on DNA methylation has emerged as a powerful molecular approach to estimate chronological age [2]. DNA methylation refers to the addition of a methyl group to DNA bases, occurring most commonly as 5-methylcytosine (5mC) at cytosine–phosphate–guanine (CpG) dinucleotides in animals, although other DNA modifications such as N6-methyladenine (6mA) have also been described [3]. Predictable, age-associated changes at specific methylation sites enable the construction of ‘epigenetic clocks’ that can estimate the age of an individual with high accuracy [4]. While this approach has been applied to several vertebrate taxa, such as cetaceans [5] and fishes [6], its application in invertebrates has been limited.

To date, invertebrate epigenetic clocks have been developed only for three arthropods: the European lobster (*Homarus gammarus*) and the jewel wasp (*Nasonia vitripennis*) using 5mC, and the buff-tailed bumblebee (*Bombus terrestris*) using 6mA [7–9]. Moreover, recent perspectives have questioned whether sparse methylation patterns and divergent life-history strategies may limit the broader applicability of epigenetic clocks in invertebrates [10]. However, DNA methylation landscapes vary widely among invertebrates, with some taxa, such as deuterostomes, exhibiting intermediate levels of genome-wide methylation [11]. Testing and developing epigenetic ageing approaches in invertebrate lineages beyond arthropods is therefore critical for assessing generality and for developing practical molecular ageing tools for ecologically important species that are otherwise difficult to age and manage.

The Pacific crown-of-thorns seastar (CoTS; *Acanthaster* cf. *solaris;* phylum Echinodermata) is a formidable and effective coral predator, and its periodic population irruptions exacerbate climate change-driven coral loss throughout the Indo-West Pacific [12]. Despite decades of study, the mechanisms underlying these irruptions remain poorly resolved [13]. A central constraint has been the lack of reliable age determination methods, limiting efforts to reconstruct recruitment timing, link cohorts to environmental conditions that facilitate outbreaks and refine population models for irruption prediction. CoTS therefore represents both an urgent coral reef management concern and a compelling opportunity to evaluate the extension of DNA methylation-based ageing from protostome to deuterostome invertebrates.

Here, we test whether CpG methylation can be used to estimate the age of CoTS through the development of an epigenetic clock. Using whole-genome methylation profiles generated across five known-age cohorts reared under controlled conditions, we identify age-associated CpG sites and construct age-predictive models. Our results demonstrate that epigenetic ageing holds significant promise for advancing age determination in deuterostome invertebrates.

## 2. Results and discussion

**Figure 1.**
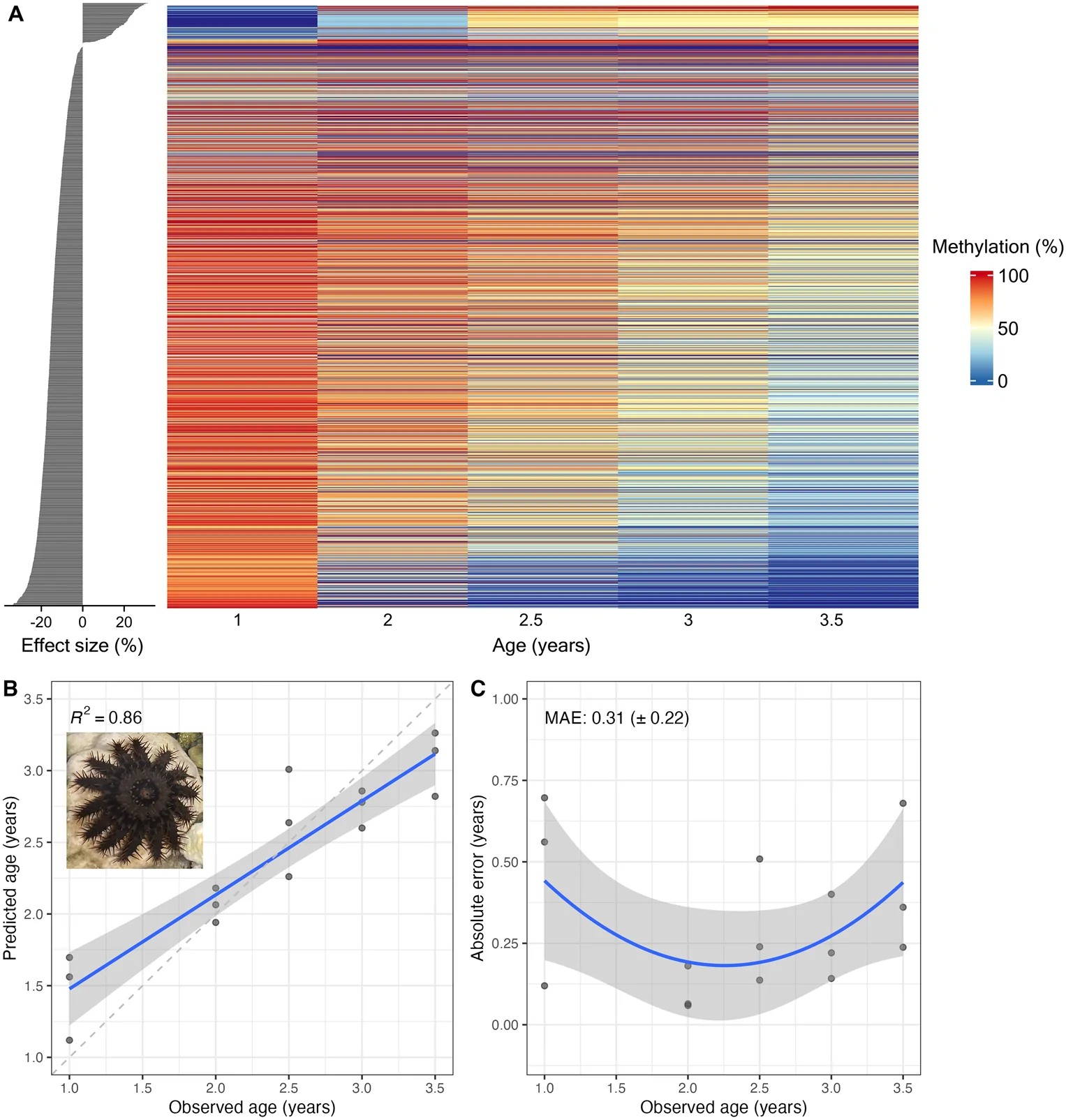
DNA methylation encodes age-associated signals in the Pacific crown-of-thorns seastar (*Acanthaster* cf. *solaris*). (A) Heatmap of methylation levels across 1910 age-associated CpG sites. The effect size plot summarizes the percentage methylation change per year. (B) Predictive performance of elastic net models evaluated using nested leave-one-out cross-validation (LOOCV; *n* = 15). (C) Absolute error of predictions across observed ages (MAE, mean absolute error). Inset: *Acanthaster* cf. *solaris.*

Age-associated CpG sites are sparse across the *A*. cf. *solaris* genome, with only 1910 sites showing significant age effects (Wald test, FDR < 0.05; figure 1A), representing 0.02% of the 9 705 479 CpG dinucleotides in the reference genome. A similarly small fraction of age-associated CpGs has been reported in other invertebrates, such as 0.03% in *Nasonia vitripennis* [8]. Fewer than 5% (83 sites) occur within CpG islands, and none were detected in rDNA regions. Consequently, reduced-representation bisulfite sequencing (RRBS), which preferentially enriches CpG-dense regions, and rDNA-based strategies, although effective in some vertebrate systems [14], are unlikely to capture sufficient age-associated signal in this and related species. Alongside prior invertebrate epigenetic clocks that relied on genome-wide methylation data [8,9], our findings suggest that future efforts to develop age-predictive models in invertebrates with similar methylation landscapes may benefit from whole-genome approaches.

We implemented elastic net regression for age prediction and evaluated model performance using leave-one-out cross-validation (LOOCV), achieving a strong correspondence between predicted and observed ages (*R*² = 0.86; figure 1B). This result demonstrates that DNA methylation can encode reliable age-associated signals in CoTS, addressing recent concerns regarding the applicability of epigenetic clocks in invertebrates [10]. Although weak or inconsistent age-related methylation signals have been reported in some taxa, such as *Daphnia magna* [15], these contrasting findings may reflect species-specific biology or methodological differences between studies and therefore cannot be generalized across invertebrates more broadly. Our successful development of an epigenetic clock for CoTS, together with prior demonstrations in lobster, wasp and bumblebee [7–9,16], provides compelling evidence that the feasibility of epigenetic clocks in invertebrates is taxa-dependent rather than universally constrained.

Error estimation indicated the potential to resolve annual age classes (mean absolute error (MAE) = 0.31 ± 0.22 years; figure 1C). This corresponds to approximately 4–6% of the estimated CoTS lifespan (5 to 8+ years) and within 9% of the maximum age tested. A fivefold cross-validation analysis yielded comparable predictive performance to LOOCV (test data MAE = 0.25 ± 0.18 years; supplementary material, figure S1). CoTS settle in annual cohorts owing to a well-defined spawning season in summer [17]; therefore, if similar predictive accuracy can be demonstrated in wild populations, this epigenetic clock could provide a valuable tool for resolving population age structure and addressing knowledge gaps related to initiation processes and population dynamics [18]. On the Great Barrier Reef (GBR), where tens of thousands of CoTS are removed annually, aggregated age profiles from culled individuals could reveal the timing and structure of outbreaks across reefs, supporting more targeted and adaptive management interventions.

From the CpGs consistently selected across LOOCV iterations (*n* = 15), we derived an epigenetic clock comprising 14 loci that probably capture key biological signals related to ageing (table 1). While predictive accuracy was evaluated using cross-validated models, this optimized clock was constructed using all available data and provides a resource for future validation and application. This reduced set of loci provides the essential information to reproduce age estimates in CoTS under comparable biological conditions. Importantly, it also offers a practical entry point for the development of targeted assays, such as multiplex bisulfite amplicon sequencing or Nanopore adaptive sequencing, facilitating cost-effective and high-throughput age determination.

**Table 1.** Details of the pacific crown-of-thorns seastar (*Acanthaster* cf. *solaris*) optimized epigenetic clock model, listing 14 age-informative CpG sites with their genomic positions, model coefficients, Pearson's correlation values (between methylation level and chronological age), and associated gene information. Gene descriptions marked with an asterisk (*) are based on BLAST analysis against deuterostomia (taxid: 33 511). The model intercept ($\beta_{0}$) is 4.5356384.

scaffold	position	model coefficient	Pearson's correlation	associated gene ID	gene description
NW_019091396.1	146 450	−0.0019125	−0.91	LOC110978098	sodium-dependent phosphate transporter 1-like, transcript variant X2
NW_019091403.1	82 170	−0.0021052	−0.92	LOC110978830	protein quaking-A-like, transcript variant X6
NW_019091406.1	902 728	−0.0052218	−0.90	LOC110979391	putative sodium-coupled neutral amino acid transporter 11, transcript variant X4
NW_019091416.1	1 359 840	−0.0016686	−0.94	LOC110980267	trinucleotide repeat-containing gene 6C protein-like, transcript variant X3
NW_019091418.1	1 514 787	−0.0050775	−0.95	LOC110980516	neuronal PAS domain-containing protein 1-like, transcript variant X1
NW_019091432.1	1 221 032	−0.0035928	−0.90	LOC110981791 LOC110981794	*glycoprotein endo-alpha-1, 2-mannosidase-like uncharacterized LOC110981794, transcript variant X4
NW_019091439.1	1 007 463	−0.0059026	−0.93	LOC110982326	clustered mitochondria protein homologue
NW_019091443.1	569 800	−0.0033427	−0.92	LOC110982539	ubiquitin carboxyl-terminal hydrolase 34-like
NW_019091457.1	403 167	−0.0029115	−0.93	LOC110983384	disintegrin and metalloproteinase domain-containing protein 12-like, transcript variant X3
NW_019091477.1	211 551	−0.0023903	−0.92	LOC110984471	PDZ and LIM domain protein 1-like, transcript variant X1
NW_019091535.1	56 884	−0.0004317	−0.92	LOC110987042	*GAS2-like protein
NW_019091655.1	169 350	−0.0002948	−0.92	LOC110989598	N(G),N(G)-dimethylarginine dimethylaminohydrolase 1-like, transcript variant X3
NW_019091851.1	16 502	−0.0102344	−0.91	LOC110990785	uncharacterized LOC110990785, transcript variant X7
NW_019091938.1	24 528	−0.0040874	−0.94	LOC110990872	uncharacterized LOC110990872

While this study establishes a foundational epigenetic clock for CoTS, future validation in wild populations is necessary. Although exact ages of wild CoTS cannot be determined, in ideal and stable environments, body size can provide coarse expectations (e.g. diameter < 200 mm probably < 2 years; > 350 mm typically > 5 years), offering an initial benchmark for field testing. Additionally, incorporating known-age individuals reared under varied environmental conditions (e.g. diet, temperature, or pH) as well as older individuals could help ensure that age-associated sites are robust to environmental variation and broaden the clock's utility, representing a substantial but valuable investment for future work.

In summary, we present an epigenetic clock for CoTS, providing a foundational molecular ageing tool with potential to inform population age structure and outbreak management. More broadly, this work demonstrates that DNA methylation-based ageing tools extend beyond arthropods, supporting their wider application in invertebrate research and management.

## 3. Material and methods

### (a) Specimen collection, DNA extraction and Nanopore sequencing workflow

Specimen collection, high molecular weight DNA extraction, Nanopore sequencing and modified basecalling were performed as described in Kwong *et al.* [11]. In brief, 12 adult CoTS (six females and six males) were collected from mid-shelf reefs in the central GBR and spawned at the Australian Institute of Marine Science (AIMS) National Sea Simulator (SeaSim) in December 2020 and 2021. The offspring were settled and raised in aquaria, with an ontogenetic dietary shift from crustose coralline algae to coral beginning at four months post-settlement. Samples were collected at 1, 2, 2.5, 3 and 3.5 years of age, each in triplicate. High molecular weight DNA was extracted from snap-frozen pyloric caeca tissue using a modified phenol–chloroform extraction protocol, followed by size selection with SPRIselect beads (Beckman Coulter). Sequencing libraries were prepared using the Native Barcoding Kit 24 V14 (SQK-NBD114-24) and sequenced on the MinION Mk1C platform implemented with MinKNOW v22.12.5, using the R10.4.1 flow cells (FLO-MIN114). Basecalling was conducted using the GPU version of Guppy v6.5.7, using the super accuracy (SUP) model dna_r10.4.1_e8.2_260bps_modbases_5mc_cg_sup. The CoTS OKI-Apl_1.0 assembly (GCF_001949145.1) was used as the reference genome.

### (b) Age-related cytosine–phosphate–guanine sites

All analyses were performed using R version 4.3.2 [19]. The methylation dataset generated from basecalling was filtered to retain CpG sites that were common across all specimens and had a minimum fivefold coverage. The methylation level of each CpG dinucleotide was defined as the proportion of methylated cytosines to the total number of reads at that site and represented as a percentage (0–100) for downstream analysis and model development. To identify age-related CpG sites, the DSS package [20] was used to fit linear models to each site, with age as the explanatory variable. DSS was supplied with the number of methylated reads (X) and the total read coverage (N) for each CpG site, allowing methylation levels to be modelled while accounting for variation in sequencing coverage across samples. The significance of the relationship between methylation level and age was assessed using the Wald test. CpG sites with a false discovery rate (FDR) < 0.05 were classified as age-related and used in downstream model development for the epigenetic clock.

Previous studies using targeted approaches have demonstrated that epigenetic clocks can be constructed by focusing on specific genomic regions. Two common strategies include targeted sequencing of rDNA regions and the use of RRBS, the latter of which enriches for CpG-rich regions, including CpG islands (short genomic regions enriched for CpG dinucleotides, often associated with gene promoters). To assess the feasibility of these targeted approaches for CoTS and related species, we examined whether the age-related sites identified in our study were located within rDNA regions or CpG islands, using the GenomicRanges package v1.54.1 [21]. Potential rDNA regions within the CoTS genome were identified through BLAST searches against rDNA sequences, including the 5S, 18S, and 28S regions of the purple sea urchin (*Strongylocentrotus purpuratus*) and the European lobster (*H. gammarus*), while CpG islands were identified using the EMBOSS cpgplot tool [22].

### (c) Epigenetic clock construction

Age prediction models were developed using an elastic net penalized regression approach, implemented with the glmnet package v4.1-8 [23]. The 1910 age-associated CpG sites identified were used as the input features for elastic net regression. To ensure reliable estimates of predictive accuracy on unseen data, an outer LOOCV approach was used to model age using DNA methylation. With this approach, each data point was sequentially held out as the test set, while the remaining data were used to train the model. This process generated 15 distinct models, each evaluated on its corresponding hold-out test point. Additionally, fivefold cross-validation was performed, following a similar process but partitioning the dataset into five parts and isolating a single part for each of five models instead of isolating individual data points. This approach evaluated the models on larger test sets, enabling comparison of model performance on train and test sets and complementing the single-point testing of LOOCV.

For both approaches, internal 10-fold cross-validation was performed to optimize the lambda parameter, which determines the strength of the penalty applied during regularization. The lambda value within one standard error of the minimum (lambda.1se) was used to balance between model complexity and performance. The alpha parameter, which controls the mixing ratio between ridge regression and LASSO regression, was set to 0.5 to balance both methods. The trained models were then used to predict the ages of the corresponding test sets. To assess the predictive performance of the models, we evaluated the relationship between the predicted and known age using Pearson's correlation. Additionally, we computed the absolute error for each prediction to assess accuracy across ages and the MAE to quantify overall prediction accuracy.

Elastic net regression performs feature selection such that during the model-building process, some CpG site coefficients were penalized to 0 and dropped from the model, retaining only the most informative sites. Common CpG sites across LOOCV iterations were identified to assess model stability and highlight those sites that contain key age-related signals. An optimized epigenetic clock was constructed using the common sites shared by all LOOCV models. As per the cross-validation steps, the lambda parameter was tuned using 10-fold inner cross-validation, and the alpha parameter was set at 0.5.

## Supplementary Material



## Data Availability

The raw sequencing data, together with the associated sample and sequencing metadata, have been deposited in the NCBI Sequence Read Archive (SRA) under BioProject accession PRJNA1429840 [24]. All analysis scripts are publicly available on Zenodo [25]. Supplementary material is available online [26].
