# Supplementary material for: DNA methylation-based ageing in a deuterostome invertebrate: an epigenetic clock for the crown-of-thorns seastar (Acanthaster cf. solaris)
Source: Biol Lett. 2026 Sep 9;22(9):20260265. doi: 10.1098/rsbl.2026.0265 (PMC13561050; doi:10.1098/rsbl.2026.0265)
Supplement: Supplementary file 1 [file rsbl.2026.0265.zip › rsbl.2026.0265/rsbl20260265_si_001.docx]

*Supplementary Information*

**DNA methylation-based ageing in a deuterostome invertebrate: an epigenetic clock for the crown-of-thorns seastar (*Acanthaster* cf. *solaris*)**

***Sarah Lok Ting Kwong ^a, b, c^	(ORCID 0000-0002-0933-9313)**

**Alyssa Maree Budd ^b, c, d, e, f^	(ORCID 0000-0002-2372-7603)**

**Maria Gomez Cabrera ^a^	(ORCID 0000-0001-9665-2123)**

**Julia Yun-Hsuan Hung ^b, c^	(ORCID 0009-0000-6809-1859)**

**Morgan Pratchett ^b^	(ORCID 0000-0002-1862-8459)**

**Cecilia Villacorta-Rath ^g^	(ORCID 0000-0002-1060-5447)**

**Sven Uthicke ^a^	(ORCID 0000-0002-3476-6595)**

a. Australian Institute of Marine Science, PMB 3 Townsville MC, Townsville, QLD 4810, Australia
b. College of Science and Engineering, James Cook University Bebegu Yumba Campus, Townsville, QLD 4811, Australia
c. Centre for Tropical Bioinformatics and Molecular Biology, James Cook University Bebegu Yumba Campus, Townsville, QLD 4811, Australia
d. Environomics Future Science Platform, Indian Ocean Marine Research Centre, Commonwealth Scientific and Industrial Research Organisation (CSIRO), Crawley, WA, Australia
e. School of Molecular and Life Sciences, Curtin University, Bentley, WA, Australia
f. The UWA Oceans Institute, The University of Western Australia, Crawley, WA, Australia
g. Centre for Tropical Water and Aquatic Ecosystem Research (TropWATER), James Cook University Bebegu Yumba Campus, Townsville, Queensland 4811, Australia

*Corresponding author; [sarah.kwong@my.jcu.edu.au](mailto:sarah.kwong@my.jcu.edu.au)

**Figure S1** Performance of elastic net models using nested 5-fold cross-validation to predict the chronological age of n = 15 Pacific crown-of-thorns seastar (*Acanthaster* cf. *solaris*). (a) Regression plot of predicted against chronological age across five cross-validation folds. (b) Box plots comparing the absolute error between training and test datasets.
